# Successful Treatment of Synchronous Double Lung Primary Malignancies and Colon Cancer

**DOI:** 10.7759/cureus.22552

**Published:** 2022-02-23

**Authors:** Hosam A Alghanmi

**Affiliations:** 1 Department of Medical Oncology, Oncology Center, King Abdullah Medical City, Makkah, SAU

**Keywords:** colon cancers, lung cancers, double primary lung cancers, synchronous colon cancer, synchronous double primary lung cancers

## Abstract

We report a rare case of multiple primary malignancies, double primary lung cancers, and synchronous colon cancer. Double primary lung cancers were synchronous with adenocarcinoma of the sigmoid (carcinoid tumor and adenocarcinoma). The patient subsequently underwent two surgeries, for lung and colon cancer, followed by four cycles of carboplatin and pemetrexed treatment for the lung adenocarcinoma, with an uneventful chemotherapy course. The patient has been followed up closely for the last 18 months via CT scans and tumor marker tests and has shown no evidence of recurrence or metastasis.

## Introduction

Multiple primary malignant tumors (MPMTs) or multiple primary carcinomas have been reported in the literature. Billroth reported multiple primary neoplasms for the first time in 1889. Later, in 1932, Warren and Gate classified MPMTs into two different types [1]. Synchronous MPMT, where a malignancy arises from a different (origin) primary tumor that develops within six months from the original primary tumor, differs from metachronous MPMT where a different tumor from the (original) primary tumor develops in more than six months after the primary tumor [2]. The international criteria of Warren and Gate MPMT are (i) each tumor must be malignant, (ii) both tumors must be different in histology, and (iii) metastasis must be excluded [3]. Here, we report synchronous double primary lung cancer with colon cancer in a patient that was discovered incidentally during staging workup, hence making it a unique case. It is crucial to rule out metastatic lung lesions from the colon, as it accounts for 20% of the cases. Further, the treatment approach will be different for such cases [4]. In our case, there were two rare entities: synchronous lung adenocarcinoma and carcinoid tumor with synchronous colon cancer. To our knowledge, no similar cases have been reported in the English literature.

## Case presentation

A 69-year-old man was diagnosed with diabetes, hypertension, hypothyroidism, and ischemic heart disease after percutaneous coronary intervention three years ago and was under follow-up in the cardiology department. He presented to the emergency department with severe abdominal pain, vomiting, and constipation. He had no family history of malignancy and was a non-smoker. Physical examination revealed abdominal distension with exaggerated bowel sounds; other systemic examinations were unremarkable. His blood function was normal.

**Figure 1:**
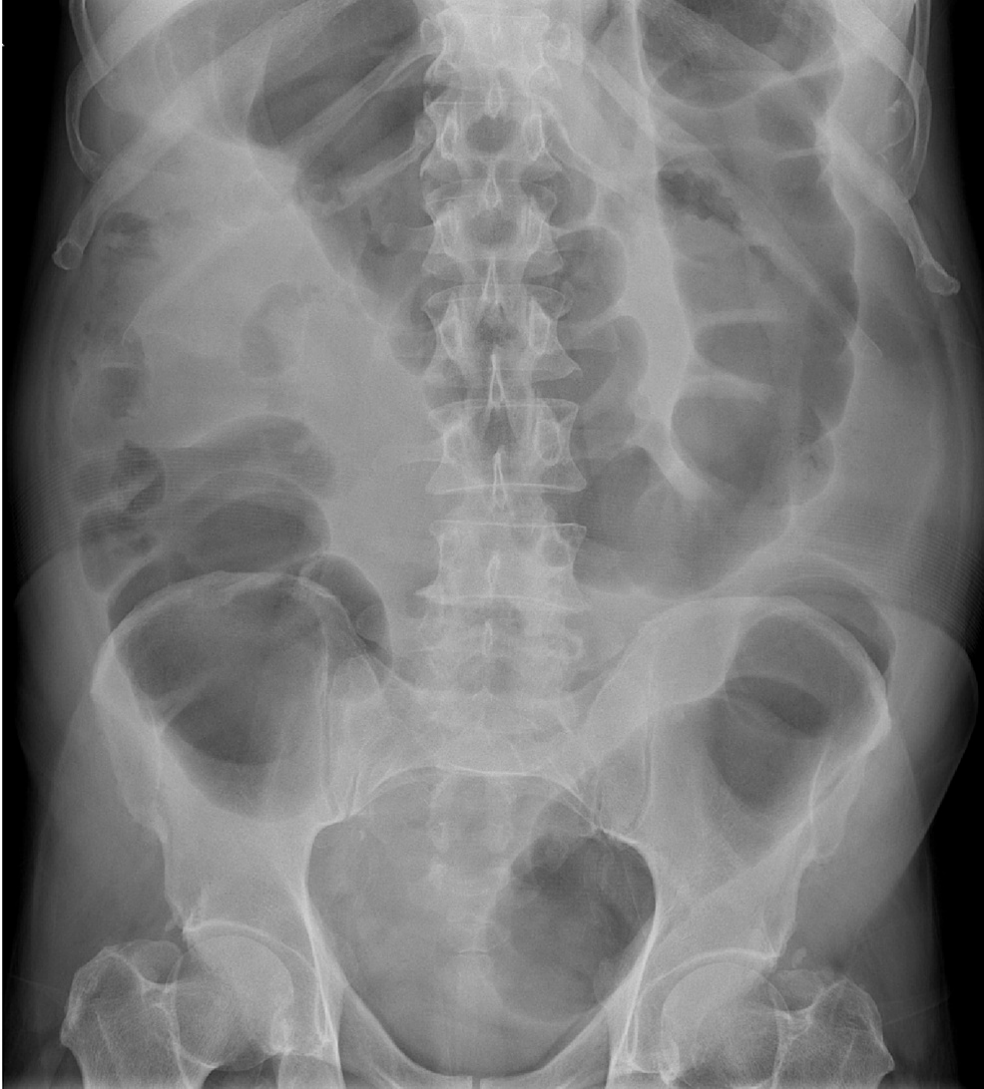
Dilated large bowel loops and multiple air-fluid.

**Figure 2:**
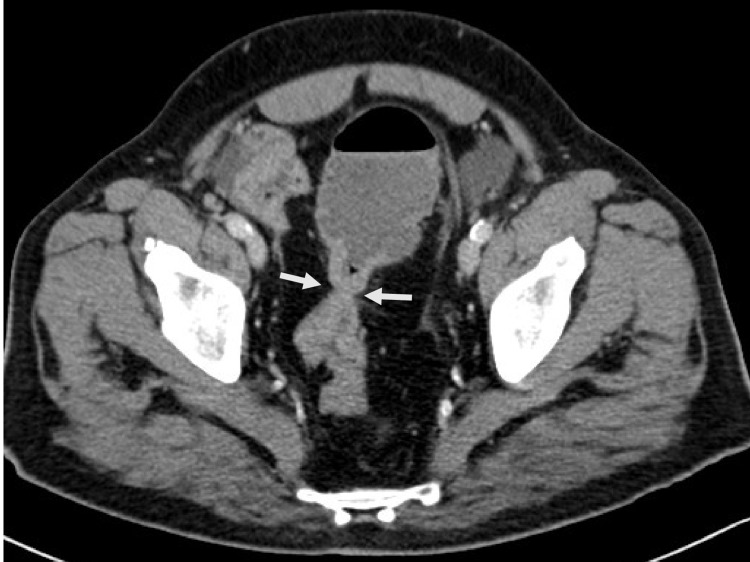
CT scan in the axial plane pelvic cut showing apple-core appearance (arrows) of the rectosigmoid mass lesion keeping with malignancy.

**Figure 3:**
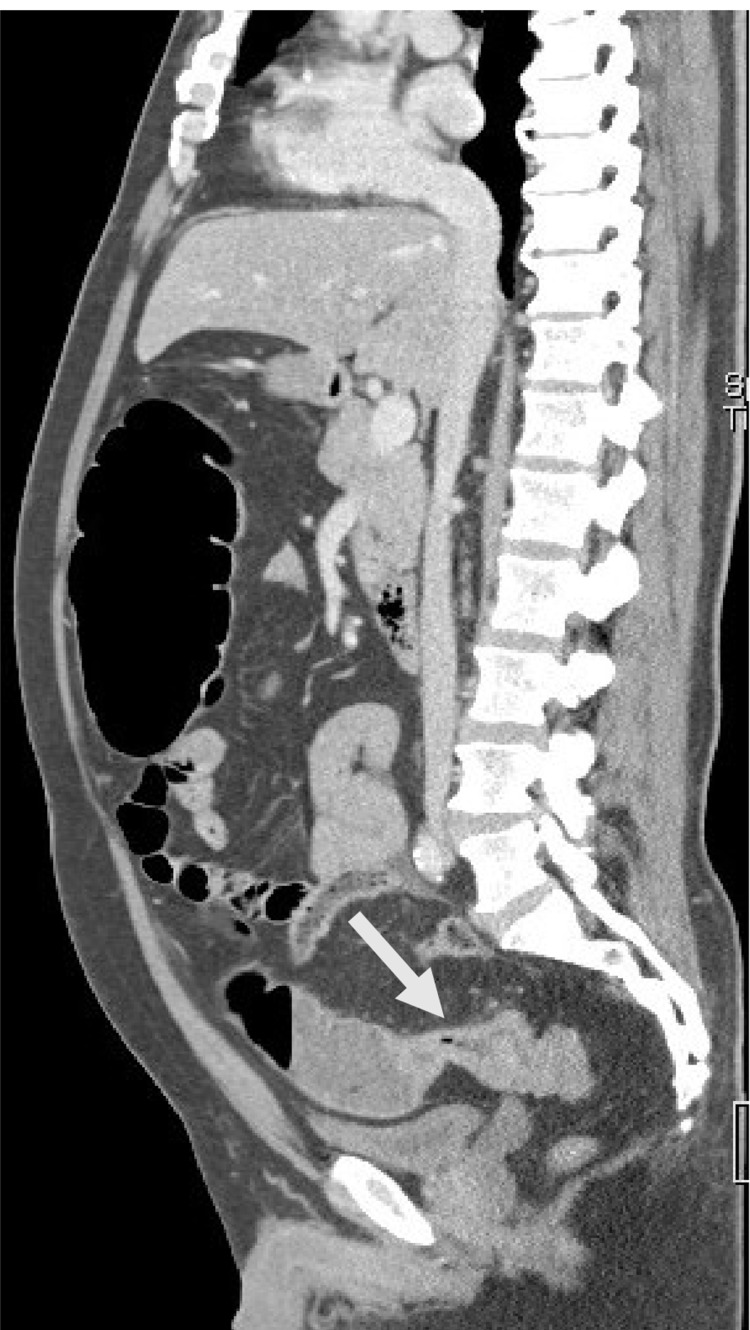
CT scan in the sagittal plane showing stricture (arrow) formed by the tumor at the rectosigmoid junction with proximal bowel dilatation.

On imaging, a standing abdominal radiograph showed dilated bowels with multiple fluid levels (Figure 1). Computed tomography (CT) of the abdomen showed evidence of circumferential wall thickening involving the rectosigmoid colon and measuring approximately 4.3 cm, associated with surrounding suspicious-looking regional lymph nodes measuring around 1 cm. Other abdominal findings were normal liver and no other intra-abdominal metastasis (Figures 2, 3).

**Figure 4:**
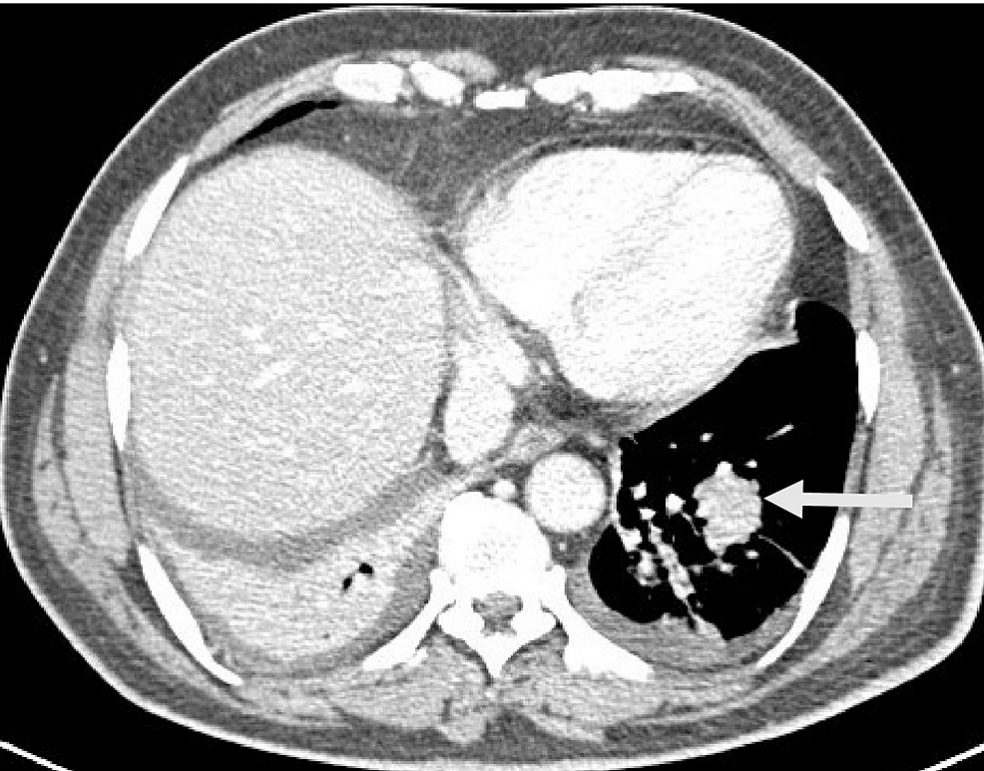
CT scan chest of the same patient’s basal cut lung window showing a left side lung mass (arrow) 2.5 cm and related atelectatic plates.

**Figure 5:**
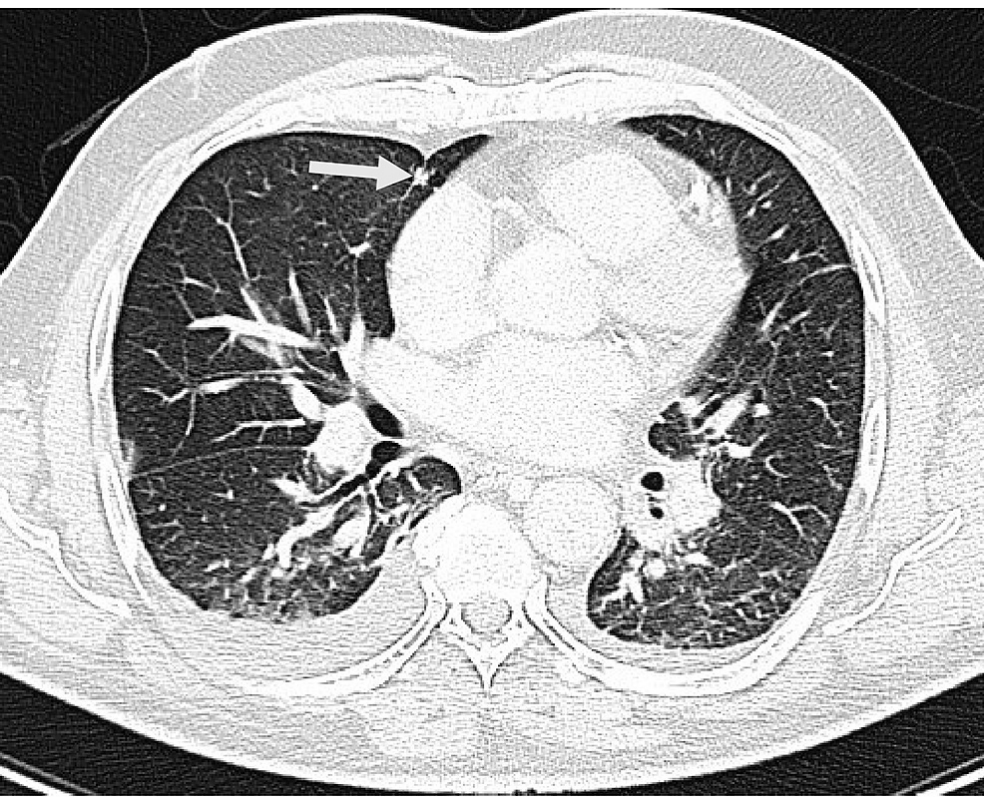
CT scan chest of the same patient’s lung window showing a small nodule (arrow) 5 mm at the middle lobe on the right side.

Visualized lower lung fields showed suspicious left lower pulmonary lesions. Chest CT showed a well-defined soft tissue nodule at the lower base of the left lower lobe measuring 2.5 cm and surrounded by atelectatic changes and sub-segmental collapse with another tiny nodule at the medial aspect of the middle lobe measuring 5 mm and mild bilateral pleural effusions (Figures 4, 5).

**Figure 6:**
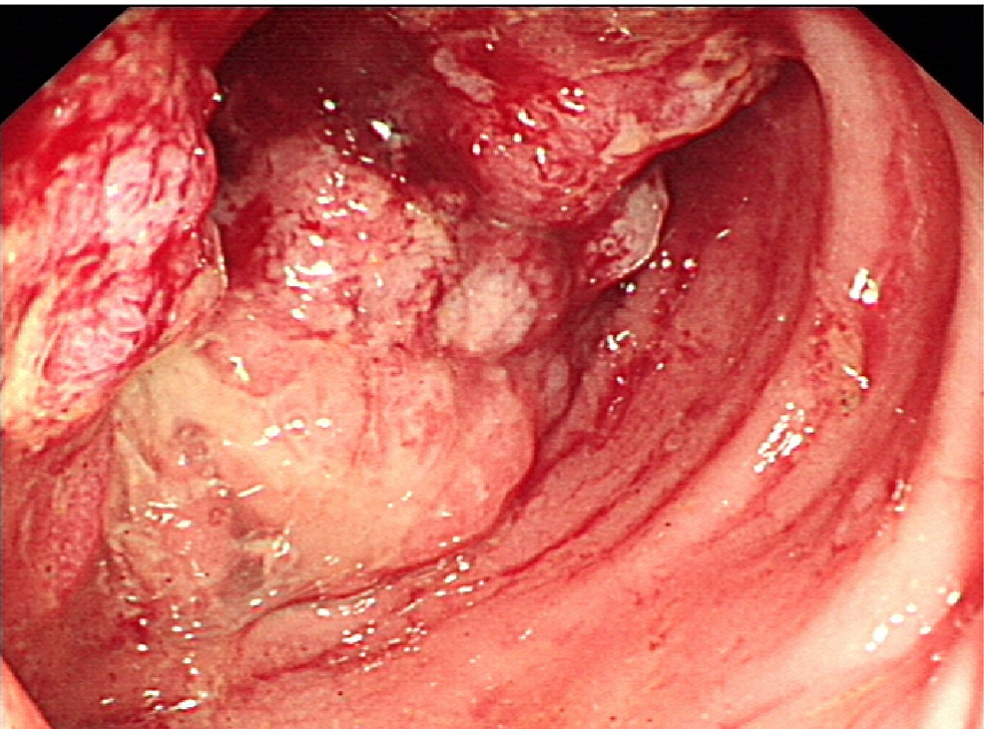
The malignant-looking mass in the rectum, 20 cm from the anus. The lesion obstructed lumen of the colon and was covered by fragile mucosa.

No significant lymphadenopathy, osteolytic bone lesions, or other suspicious findings were observed. The patient was admitted to the surgical department for further management and underwent diversion colostomy by a colorectal surgery team. Gastroenterological and thoracic surgery consultations were performed. One week later, the patient underwent colonoscopy, which showed a circumferential fungating malignant-like mass 20 cm from the anus, easily bleeding with narrowing of the lumen. We obtained multiple biopsies (Figure 6).

Histopathological results confirmed the presence of adenocarcinoma of the sigmoid colon. Immunohistochemistry of sigmoid mass showed cytokeratin (CK)20 and caudal type homeobox 2 (CDX2), positive in tumor cells. CT-guided biopsy of the lung nodule in the left lower lobe showed adenocarcinoma of the lung. Immunohistochemistry of lung lesions showed (CK7, CK19, and thyroid transcription factor 1 {TTF-1}: strong and diffuse positive in tumor cells, and CK20 and CDX2: negative in tumor cells).

Complete blood count, renal function, and liver function were normal while carcinoembryonic antigen (CEA) was high. Abdominal x-ray showed multiple air-fluid levels, which indicate presence of intestinal obstruction. Computed tomography (CT) of chest, abdomen, and pelvis showed two lung lesions and a rectosigmoid lesion.

A colonoscopy showed a malignant-looking mass 20 cm from the anal verge that could easily bleed with narrowing of the lumen. Biopsy results from sigmoid colon mass confirmed the presence of adenocarcinoma of sigmoid colon, and histopathology results from the two lung lesions confirmed adenocarcinoma of the lung in one lesion and confirmed a low-grade neuroendocrine tumor in the other lesion.

The tumor board recommended resection of the primary lung cancer, followed by colon cancer surgery. Three weeks later, the patient underwent left lower lobectomy with wedge resection of the right middle lobe lesions, and the left lower lesion was confirmed adenocarcinoma of primary lung origin with pathological stage (pT1c, pN1, pMx), corresponding to stage IIB based on the 8th edition of the American Joint Committee on Cancer (AJCC) [5]. The right middle lobe lesion interestingly showed a low-grade neuroendocrine tumor (positive for synaptophysin and chromogranin A with a Ki-67 labeling index of approximately 2% and a mitotic count of 2/10 HPF) with pathological stage T1a, N0, Mx, corresponding to stage IA based on the 8th edition of the tumor, nodes, and metastases (TNM) staging [6].

Outcomes and follow-up

A month later, after recovery from lung surgery, the patient was seen by the colorectal surgery team, and he underwent sigmoidectomy with anastomosis. The histopathology report revealed a moderately differentiated adenocarcinoma invading the muscularis propria with negative margins without lymph vascular invasion or perineural invasion with one positive lymph node out of 14 that were resected. The histopathological stage was T2, N1a, and Mx, corresponding to stage IIIA, based on the 8th edition of TNM staging [6]. One month after colon surgery, CT scans showed no evidence of residual tumor recurrence. The patient was then seen in the medical oncology clinic and the adjuvant chemotherapy was completed for lung adenocarcinoma. The patient received four cycles of carboplatin with pemetrexed with good tolerance. Follow-up of the patient in the clinic with images and tumor markers performed one year after both surgeries showed no local recurrence or metastasis. The surgical team performed closure of colostomy, and the patient is currently in good health with regular follow-up with frequent imaging and tumor marker analysis.

## Discussion

Multiple primary tumors, based on the National Cancer Institute and Surveillance, Epidemiology, and End Results (SEER) data, account for 8% of cancer cases in the United States [7]. Although the development of MPMT is unclear, there are some risk factors such as smoking, genetics, advanced age, diet, and previous malignancy [8]. Prolongation of age due to advancements in disease treatment procedures has led to an increased occurrence of MPMT (5% of all malignant cancer cases) [9]. Another data-based analysis of 50,000 patients over 40 years found that the incidence of more than one tumor in one patent was 0.1% [7]. There are multiple retrospective studies, one from China, revealing the incidence to be 1.09% of the study population, and another from Turkey, between 2003 and 2004, confirming the incidence rate to be 1.2% [3,10]. The incidence of primary lung cancer with colon cancer is very rare. Based on data from Southeast England (2018), synchronous colon and lung cancers accounted for 0.6% of the total 127,281 patients treated in multiple centers, which is considered extremely rare [11]. In other reports of double primary lung cancers, adenocarcinoma and bronchial carcinoid tumors are considered very rare with an incidence of less than 1% and a maximum incidence rate of 16% [11]. In the literature review, most reported cases of double primary tumors of the lung and colon are either single lung neoplasms with single colon neoplasms [3,4,12] or double lung primary neoplasms [13].

A review of literature in databases (in English) such as PubMed, ResearchGate, and Google Scholar, revealed no case similar to ours. There is a paucity of literature on the management of such cases. Different methods are needed to confirm synchronous cancer such as biopsy with immunohistochemistry. Patients with a history of multiple cancers are usually excluded from the clinical setting; therefore, there is no clear evidence regarding their treatment [3]. The treatment of such patients requires multidisciplinary teams. A suggested approach for localized cancer may include chemotherapy or chemoradiotherapy [14,15]. Important points to be considered with synchronous malignancies are patient fitness, prognosis of each cancer, chance of cure, whether one tumor can be treated radically and the other sequentially, anticipated complications, obstructive symptoms, and if other similar treatments can be used [16]. In our case, the treatment initially focused on the acute presentation of the patient, and thus, diversion colostomy was prioritized. Two surgeries were sequentially performed with a one-month interval for synchronous cancers. Both synchronous cancers require adjuvant treatment, and the chemotherapy strategies are different. The panelists recommended treating the most aggressive cancer, which was lung adenocarcinoma. Based on the study of outcomes of synchronous lung cancer and colon cancer in 17 patients, lung cancer was considered as a poor prognostic factor and the cause of death among patients. Regarding low-grade neuroendocrine tumors of the lung, the patient was asymptomatic and underwent resection, based on the National Comprehensive Cancer Network (NCCN) guideline [17]. Different treatment approaches have been used on a case-by-case basis and depending on the experience of the doctors at the cancer center. Based on previous case reports, some patients underwent two surgeries for both primary cancers and chemotherapy. As there is no standard of care, a multidisciplinary team approach is needed for the best patient care.

## Conclusions

Double primary lung cancers were synchronous with adenocarcinoma of the sigmoid (carcinoid tumor and adenocarcinoma) is a rare case. A multidisciplinary approach is essential for treating oncology patients, especially in complications like MPMT where no standard of care treatment is established. In case of MPMT and turned to need adjuvant chemotherapy, we should start with the most aggressive cancer in adjuvant setting. 
